# The Global Diet Quality Score Is Inversely Associated with Nutrient Inadequacy, Low Midupper Arm Circumference, and Anemia in Rural Adults in Ten Sub-Saharan African Countries

**DOI:** 10.1093/jn/nxab161

**Published:** 2021-10-23

**Authors:** Sabri Bromage, Yiwen Zhang, Michelle D Holmes, Sonia E Sachs, Jessica Fanzo, Roseline Remans, Jeffrey D Sachs, Carolina Batis, Shilpa N Bhupathiraju, Teresa T Fung, Yanping Li, Meir J Stampfer, Megan Deitchler, Walter C Willett, Wafaie W Fawzi

**Affiliations:** Harvard T.H. Chan School of Public Health, Boston, MA, USA; Harvard T.H. Chan School of Public Health, Boston, MA, USA; Harvard T.H. Chan School of Public Health, Boston, MA, USA; Channing Division of Network Medicine, Brigham & Women's Hospital, Boston, MA, USA; The Earth Institute, Columbia University, New York, NY, USA; Berman Institute of Bioethics, Nitze School of Advanced International Studies, Johns Hopkins University, Baltimore, MD, USA; The Alliance of Biodiversity International and the International Center for Tropical Agriculture (CIAT), Geneva, Switzerland; The Earth Institute, Columbia University, New York, NY, USA; CONACYT—Health and Nutrition Research Center, National Institute of Public Health, Cuernavaca, Mexico; Harvard T.H. Chan School of Public Health, Boston, MA, USA; Channing Division of Network Medicine, Brigham & Women's Hospital, Boston, MA, USA; Harvard T.H. Chan School of Public Health, Boston, MA, USA; Department of Nutrition, Simmons University, Boston, MA, USA; Harvard T.H. Chan School of Public Health, Boston, MA, USA; Harvard T.H. Chan School of Public Health, Boston, MA, USA; Channing Division of Network Medicine, Brigham & Women's Hospital, Boston, MA, USA; Intake – Center for Dietary Assessment, FHI Solutions, Washington, DC, USA; Harvard T.H. Chan School of Public Health, Boston, MA, USA; Channing Division of Network Medicine, Brigham & Women's Hospital, Boston, MA, USA; Harvard T.H. Chan School of Public Health, Boston, MA, USA

**Keywords:** diet quality metrics, dietary diversity, nutrient adequacy, noncommunicable disease, double burden of malnutrition, nutrition transition, nutritional epidemiology, Millennium Villages Project, sub-Saharan Africa, GDQS

## Abstract

**Background:**

Key nutrient deficits remain widespread throughout sub-Saharan Africa (SSA) whereas noncommunicable diseases (NCDs) now cause one-third of deaths. Easy-to-use metrics are needed to track contributions of diet quality to this double burden.

**Objectives:**

We evaluated comparative performance of a novel food-based Global Diet Quality Score (GDQS) against other diet metrics in capturing nutrient adequacy and undernutrition in rural SSA adults.

**Methods:**

We scored the GDQS, Minimum Dietary Diversity–Women (MDD-W), and Alternative Healthy Eating Index–2010 (AHEI-2010) using FFQ data from rural men and nonpregnant, nonlactating women of reproductive age (15–49 y) in 10 SSA countries. We evaluated Spearman correlations between metrics and energy-adjusted nutrient intakes, and age-adjusted associations with BMI, midupper arm circumference (MUAC), and hemoglobin in regression models.

**Results:**

Correlations between the GDQS and an energy-adjusted aggregate measure of dietary protein, fiber, calcium, iron, zinc, vitamin A, folate, and vitamin B-12 adequacy were 0.34 (95% CI: 0.30, 0.38) in men and 0.37 (95% CI: 0.32, 0.41) in women. The GDQS was associated (*P* < 0.05) with lower odds of low MUAC [GDQS quintile (Q) 5 compared with Q1 OR in men: 0.44, 95% CI: 0.22, 0.85; women: 0.57, 95% CI: 0.31, 1.03] and anemia (Q5/Q1 OR in men: 0.56, 95% CI: 0.32, 0.98; women: 0.60, 95% CI: 0.35, 1.01). The MDD-W correlated better with some nutrient intakes, though associated marginally with low MUAC in men (*P* = 0.07). The AHEI-2010 correlated better with fatty acid intakes, though associated marginally with low MUAC (*P* = 0.06) and anemia (*P* = 0.14) in women. Overweight/obesity prevalence was low, and neither the GDQS, MDD-W, nor AHEI-2010 were predictive.

**Conclusions:**

The GDQS performed comparably with the MDD-W in capturing nutrient adequacy–related outcomes in rural SSA. Given limited data on NCD outcomes and the cross-sectional study design, prospective studies are warranted to assess GDQS performance in capturing NCD outcomes in SSA.

## Introduction

Traditional diets of sub-Saharan Africa (SSA) were largely plant-based, emphasizing fruits and vegetables, legumes, wild cereals, roots and tubers, and supplemented with fish, dairy, and modest amounts of game, poultry, and red meat (1). Diets shifted dramatically following colonial incursions, the introduction of maize in the 1500s and its later emergence as the dominant staple throughout SSA (2), and recent decades of increasing incomes, urbanization, and food market globalization (3). From 2001 to 2018, the prevalence of inadequate energy intakes in SSA fell from 27.3% to 21.4%, but it remains among the highest of world regions (4). Dietary shifts have also led to increased consumption of obesogenic processed foods and refined carbohydrates, which have replaced traditional and more nutrient-dense foods (5, 6). The regional food supply currently contains the lowest percentage of calories supplied per capita by protein-rich animal-source foods (8.2%) globally (7). Countries in SSA also face the highest burden of hidden hunger globally (defined in terms of disability-adjusted life years collectively attributed to iron, vitamin A, and zinc deficiencies) (8), and the second highest prevalence of child stunting and wasting after South Asia (9).

SSA is also undergoing a steady epidemiological transition toward noncommunicable diseases (NCDs) (6, 10–12), and most of the world's countries with coexisting burdens of stunting, anemia, and overweight are currently in Africa (13, 14). The increasing prevalence of overweight is driven predominantly by the urban population [unlike other world regions, rural obesity rates in SSA are still lagging compared with urban areas (15)]. Since 2000 alone, the fraction of total mortality contributed by NCDs in SSA increased from 22.7% to 32.6% (16); hypertension and dyslipidemia are common (17–19); and the regional prevalence of type 2 diabetes is uncertain but evidently increasing (20). At present, diet contributes a smaller percentage of age-standardized cardiovascular, cancer, and type 2 diabetes mortality in Central (15%), Eastern (14%), Western, and Southern (13%) SSA than any other world region (21), and consumption of dietary components associated with NCD risk—red meat, sugar, saturated and *trans* fat, and sodium—is relatively low (21–23). However, the age-standardized fraction of mortality attributable to dietary risks of NCDs in SSA has increased in 42 of 51 SSA countries from 1990 to 2015 (from 10.4% to 12.2% in the region overall) (21, 24), and diet quality [measured using the Alternative Healthy Eating Index–2010 (AHEI-2010)] has deteriorated more in SSA than in other regions from 1990 to 2017 (25).

In light of persisting undernutrition throughout SSA, evidence that the increasing burden of NCDs is outpacing reductions in child and maternal malnutrition (24, 26), and the likelihood that the regional NCD burden will increase significantly as lifespans continue to lengthen, multiple coexisting burdens of malnutrition will continue to pose a major threat to the future of public health in SSA. Given the added context of worsening diet quality in the region, it is especially important that SSA has the tools needed to measure and track diet quality in terms of both dietary nutrient adequacy and NCD risk. Easy-to-use, food-based metrics are particularly attractive given the region's limited resources for conducting diet surveys and limited national food composition data with which to compute nutrient intakes (27, 28).

In this article we describe a secondary analysis evaluating the performance of a novel food-based metric, the Global Diet Quality Score (GDQS) (29) for predicting diet quality outcomes in rural men and women living in 10 SSA countries participating in the Millennium Villages Project (MVP), and we compare the performance of the GDQS with that of existing diet metrics.

## Methods

### Study population

We analyzed data from the MVP (30, 31). The MVP was a multiyear sustainable development project conducted from 2004 to 2015 in 14 rural villages located in 10 sub-Saharan African countries. The current analysis included data collected from men and nonpregnant nonlactating women of reproductive age (15–49 y) living in 12 Millennium Villages in 10 countries: Koraro (Ethiopia), Bonsaaso (Ghana), Dertu and Sauri (Kenya), Mwandama (Malawi), Tiby (Mali), Ikaram and Pampaida (Nigeria), Mayange (Rwanda), Potou (Senegal), Mbola (Tanzania), and Ruhiira (Uganda). We analyzed data from the first 2 waves of evaluations, which include data from 2005 to 2010 and every calendar month. We pooled both waves of data in all analyses. Although the MVP included panel measurements, we did not analyze data longitudinally given the challenge of adequately controlling for the influence of multiple large-scale community interventions implemented as part of the project; these interventions collectively brought about broad nutritional improvements that could confound associations between diet metrics and outcomes. This analysis was approved by the Institutional Review Boards of Columbia University and Harvard T.H. Chan School of Public Health.

### Dietary assessment

Diet was assessed from each participant using nonquantitative FFQs specifically tailored to each country to capture local food consumption. All FFQs used a reference period of the last 1 mo and the following frequency response categories for all foods: never, 1/mo, 2–3/mo, 1/wk, 2–3/wk, 4–6/wk, 1/d, ≥2/d. The number of foods assessed by each FFQ ranged from 92 to 161.

We derived standard portion sizes for each food through analysis of quantitative 24-h recall (24HR) survey data collected from women of reproductive age in Burkina Faso, Ethiopia, Uganda, and Zambia (32, 33), in which we grouped similar foods together and computed the median daily consumed mass of each food or food group. The analyzed 24HR surveys sampled women of reproductive age regardless of pregnancy or breastfeeding status, and these surveys included some pregnant or lactating women; we retained these women in our analysis to ensure adequate statistical power for deriving standard portion sizes for less frequently consumed foods. In addition to computing standard portions in the pooled population of 4 countries, we computed country-specific standards for Ethiopia and Uganda, which we supplemented with published serving sizes for adults from Nigeria, Tanzania, and Uganda (34, 35), to allow us to match foods, where possible, on a country-by-country basis with foods consumed in the MVP data.

We computed intakes of a set of nutrients we considered high priority in low- and middle-income countries: protein, monounsaturated fat, polyunsaturated fat, saturated fat, dietary fiber, calcium, iron, zinc, vitamin A, folate, and vitamin B-12. The primary source for nutrient composition of foods was the 2008 Food Composition Tables (FCTs) for Tanzania (36), including >400 foods (most of which are also consumed outside of Tanzania) and detailed data on dietary fatty acids. For consumed foods missing from the Tanzania FCT, we abstracted data from other African FCTs that also distinguished fatty acid fractions (Kenya, Senegal, Mozambique, and Egypt) (37, 38), and from the United States and Germany (for certain internationally available packaged foods) (39, 40). In combining food composition data from multiple countries, we rendered them compatible by adjusting nutrients as appropriate for differences in moisture and fat content according to FAO guidelines (41). Because each FFQ collected data primarily at the level of ingredients, calculation of recipes was not necessary, whereas some cooking yield and nutrient retention factors were drawn from international references and applied to raw ingredients (42–45).

### Scoring diet metrics

For both men and women, FFQ data were used to tabulate the following metrics [refer to the article by Bromage et al. (29) introducing this Supplement for information on how these metrics are constructed and scored]:

- Food-based metrics intended to reflect overall diet quality: the GDQS (29) and a Prime Diet Quality Score (PDQS)-like metric, an adaptation of an earlier metric (the PDQS) (46–48) from which the GDQS was developed.
- Food-based metrics intended to reflect nutrient adequacy: the GDQS-positive submetric (GDQS+) (29) computed using only the healthy GDQS food groups, and the Minimum Dietary Diversity–Women indicator (MDD-W) (49). We acknowledged that the MDD-W was originally intended for use in women only; furthermore, we treated this metric as a continuous integer variable ranging from 0 to 10, rather than as a binary indicator as it is sometimes used.
- Metrics intended to reflect NCD risk: the GDQS-negative submetric (GDQS−) (29)computed using only the unhealthy GDQS food groups, and the AHEI-2010 (50) scored using both food and nutrient components.

### Diet quality outcomes

We estimated energy-adjusted nutrient intakes using the residual method (51). We constructed a continuous measure of overall nutrient adequacy based on the number of nutrients (out of 8) meeting age- and sex-specific estimated average requirements (EARs) from the Institute of Medicine (or adequate intake level, in the case of fiber) (52); iron adequacy was defined as ≥50% probability of adequacy based on a log normal requirement distribution (53). Iron requirement distributions and zinc EARs were adjusted to account for absorption characteristics of local diets (53–56). We also created a binary measure of overall nutrient inadequacy using a cutoff of <4 (out of 8) adequate nutrients, as well as energy-adjusted continuous measures of overall nutrient adequacy and binary overall nutrient inadequacy.

In addition to nutrient intake and adequacy, we also analyzed data on BMI (kg/m^2^); midupper arm circumference (MUAC); and hemoglobin collected from a subsample of participants using standard laboratory procedures available in each country (adjusted for the altitude of each village) (57) in men and women. The following cutoffs were applied to derive binary outcomes:

- Underweight and overweight/obesity: BMI <18.5 and ≥25 in both men and women (58)
- Low MUAC: <25.5 cm in men and <24.5 cm in women; these cutoffs were identified as those resulting in the lowest overall misclassification of underweight BMI in an international analysis (59)
- Anemia: <13 g/dL in men and <12 g/dL hemoglobin in women (altitude-adjusted) (57)

### Analysis of metric performance

We evaluated and compared the performance of the GDQS, GDQS+, GDQS−, PDQS-like metric, MDD-W, and AHEI-2010 against diet quality outcomes. Methods involved computing Spearman correlations between metrics and continuous diet quality outcomes; regression models to determine unadjusted and age-adjusted estimated marginal means or ORs for different diet quality outcomes within each metric quintile and in terms of a 1 SD difference in each metric; and statistical comparisons of correlation coefficients, and trends in measures of association across quintiles, between pairs of metrics (50, 60).

We excluded women who indicated they were currently pregnant or lactating. Within each sex, we also excluded participants with no reported food consumption, followed by participants with energy intakes <3 or >3 SDs from the mean, to limit the influence of implausible values. Correlation and regression analyses were performed separately in the total population (i.e., pooled across villages) of men and the total population of women. For the GDQS alone, within pooled men and pooled women we also determined partial correlations with energy-adjusted nutrients controlling for village. In correlating metrics and energy-adjusted iron intakes in pooled analyses across villages, we excluded participants from Ethiopia, whose iron intakes were extremely high compared with those of other countries owing to the contribution of teff. In addition to correlation analyses pooled across villages, for all metrics we also derived correlations within each village (in doing so, we pooled men and women within each village to optimize sample size).

In interpreting comparative metric performance, we prioritized correlations with energy-adjusted nutrient intakes and age-adjusted regression models rather than unadjusted results, and defined a subset of higher relevance diet quality outcomes in regression models (the continuous measure of energy-adjusted overall nutrient adequacy, and outcomes defined using clinically relevant cutoffs: overweight, underweight, low MUAC, and anemia) distinguished from lower relevance outcomes (the binary measure of energy-adjusted overall nutrient inadequacy and continuous outcomes for which clinically relevant cutoffs exist: BMI, MUAC, and hemoglobin).

Statistical analyses were performed in R version 4.03 (R Foundation).

## Results

FFQ data from 1547 men and 1624 nonpregnant nonlactating women of reproductive age were analyzed in this study (age range = 15–49 y and median = 30 y for both groups). Descriptive statistics on the number of participating men and women by village and survey wave, prevalence of binary diet quality outcomes by sex, and distributions of GDQS food group consumption and metric scores by sex and village are provided in **Supplemental Tables 1–4**. Pooling across villages, mean GDQS scores did not differ between men (22.6 ± 4.4) and women (22.5 ± 3.4) (*P* ≥ 0.05) (Supplemental Table 4). Within villages, pooling men and women, the lowest mean GDQS score was found in Dertu, Kenya (18 ± 2.5) and the highest in Pampaida, Nigeria (26.3 ± 3.4). Correlations between the number of foods listed in the FFQs for each village compared with mean metric scores in each village were nonsignificant (*P* ≥ 0.05) (**Supplemental Table 5**).

In pooled analysis of all villages, the GDQS was significantly (*P* < 0.05) and at least modestly rank-correlated with energy-adjusted intakes of fiber (men: *r* = 0.22/women: 0.25), folate (0.13/0.24), monounsaturated fat (0.24/0.28), polyunsaturated fat (0.12/0.12), and vitamin A (0.10/0.15) (**Table 1**). In women, we also observed a modest correlation with protein (*r* = 0.14), and a negative correlation with zinc (−0.11). The GDQS was weakly (*r* < 0.1) or nonsignificantly (*P* ≥ 0.05) correlated with energy-adjusted calcium, iron, saturated fat, and vitamin B-12 intakes in men and women.

**FIGURE 1:**
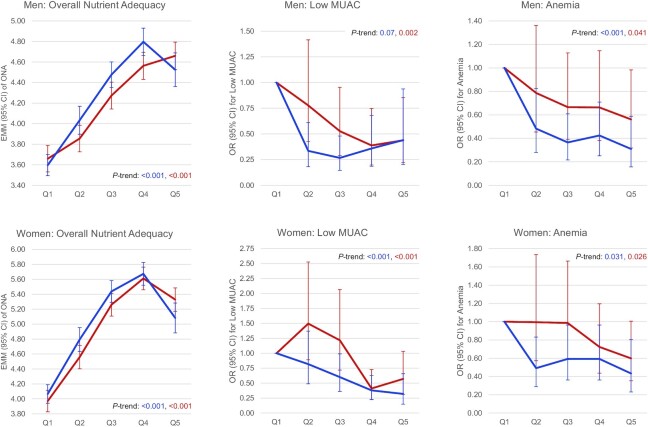
Age-adjusted associations between quintiles of the GDQS (red) and MDD-W (blue) compared with overall nutrient adequacy, low midupper arm circumference (MUAC), and anemia in rural sub-Saharan African adults. Overall nutrient adequacy (ONA) defined as energy-adjusted number of adequate nutrients (out of 8). Low MUAC defined as <25.5 cm in men and <24.5 cm in women. Anemia defined as <13 g/dL in men and <12 g/dL in women (altitude-adjusted). GDQS quintiles correspond to scores <18.8,  18.9–21.3, 21.3–23.5, 23.5–26.5, and >26.5 in men; and <19.0,  19.0–21.3, 21.3–23.5, 23.5–26.3, and >26.3 in women. MDD-W quintiles correspond to scores <5,  5.0, 6.0, 7.0, and >7 in men and women. All Wald tests comparing linear trends across quintiles between the GDQS and MDD-W were nonsignificant (*P* ≥ 0.05). EMM, estimated marginal mean, GDQS, Global Diet Quality Score; MDD-W, Minimum Dietary Diversity–Women; Q, quintile.

**TABLE 1:** Comparison of Spearman correlations between metrics (scored using FFQ data) and energy-adjusted nutrients and clinical measurements in rural sub-Saharan African adults^1^

			GDQS	GDQS+		GDQS−		PDQS-like metric		MDD-W		AHEI-2010	
Outcome	Sex	*n*	*r*	*r*	*P*-difference	*r*	*P*-difference	*r*	*P*-difference	*r*	*P*-difference	*r*	*P*-difference
Calcium	M	1547	−0.04	−0.10^2^	<0.001^2^	0.17^2^	<0.001^2^	−0.05	0.44	−0.05^2^	0.46	−0.06^2^	0.31
	F	1624	−0.02	−0.11^2^	<0.001^2^	0.23^2^	<0.001^2^	−0.09^2^	<0.001^2^	−0.05	0.20	−0.05^2^	0.33
Fiber intake	M	1547	0.22^2^	0.30^2^	<0.001^2^	−0.29^2^	<0.001^2^	0.19^2^	0.53	0.20^2^	0.44	0.25^2^	0.11
	W	1624	0.25^2^	0.32^2^	<0.001^2^	−0.25^2^	<0.001^2^	0.19^2^	0.008^2^	0.28^2^	0.041^2^	0.30^2^	0.07
Folate	M	1547	0.13^2^	0.21^2^	<0.001^2^	−0.28^2^	<0.001^2^	0.04	<0.001^2^	0.13^2^	0.87	0.10^2^	0.42
	W	1624	0.24^2^	0.33^2^	<0.001^2^	−0.29^2^	<0.001^2^	0.16^2^	<0.001^2^	0.25^2^	0.53	0.22^2^	0.25
Iron intake	M	1422	0.02	0.05	<0.001^2^	−0.13^2^	<0.001^2^	−0.02	0.43	−0.08^2^	<0.001^2^	−0.08^2^	<0.001^2^
	W	1410	0.02	0.07^2^	<0.001^2^	−0.13^2^	<0.001^2^	−0.01	0.59	−0.05	<0.001^2^	−0.08^2^	<0.001^2^
MUFA	M	1547	0.24^2^	0.20^2^	0.001^2^	0.05^2^	<0.001^2^	0.15^2^	<0.001^2^	0.26^2^	0.32	0.29^2^	0.039^2^
	W	1624	0.28^2^	0.27^2^	0.76	−0.05	<0.001^2^	0.20^2^	<0.001^2^	0.33^2^	0.018^2^	0.32^2^	0.12
Protein	M	1547	0.07^2^	0.02	0.001^2^	0.08^2^	0.63	−0.06^2^	<0.001^2^	0.02	0.020^2^	−0.11^2^	<0.001^2^
	W	1624	0.14^2^	0.07^2^	<0.001^2^	0.11^2^	0.59	0.00	<0.001^2^	0.07^2^	<0.001^2^	−0.02	<0.001^2^
PUFAs	M	1547	0.12^2^	0.06^2^	<0.001^2^	0.13^2^	0.82	0.19^2^	<0.001^2^	0.11^2^	0.87	0.37^2^	<0.001^2^
	W	1624	0.12^2^	0.06^2^	<0.001^2^	0.10^2^	0.58	0.16^2^	0.014^2^	0.11^2^	0.51	0.29^2^	<0.001^2^
SFAs	M	1547	0.02	0.09^2^	<0.001^2^	−0.16^2^	<0.001^2^	−0.11^2^	<0.001^2^	0.08^2^	0.004^2^	−0.13^2^	<0.001^2^
	W	1624	0.05^2^	0.13^2^	<0.001^2^	−0.19^2^	<0.001^2^	−0.02	<0.001^2^	0.09^2^	0.09	−0.11^2^	<0.001^2^
Vitamin A	M	1547	0.10^2^	0.21^2^	<0.001^2^	−0.28^2^	<0.001^2^	0.22^2^	<0.001^2^	0.22^2^	<0.001^2^	0.21^2^	<0.001^2^
	W	1624	0.15^2^	0.26^2^	<0.001^2^	−0.32^2^	<0.001^2^	0.27^2^	<0.001^2^	0.25^2^	<0.001^2^	0.20^2^	0.07
Vitamin B-12	M	1547	−0.03	−0.04	0.68	0.04	0.030^2^	0.00	0.07	−0.01	0.17	−0.04	0.84
	W	1624	0.09^2^	0.11^2^	0.09	−0.05	<0.001^2^	0.10^2^	0.54	0.12^2^	0.15	0.03	0.029^2^
Zinc	M	1547	−0.02	−0.12^2^	<0.001^2^	0.20^2^	<0.001^2^	−0.12^2^	<0.001^2^	−0.15^2^	<0.001^2^	−0.27^2^	<0.001^2^
	W	1624	−0.11^2^	−0.22^2^	<0.001^2^	0.28^2^	<0.001^2^	−0.21^2^	<0.001^2^	−0.20^2^	<0.001^2^	−0.25^2^	<0.001^2^
ONA	M	1547	0.34^2^	0.38^2^	<0.001^2^	−0.15^2^	<0.001^2^	0.25^2^	<0.001^2^	0.37^2^	0.13	0.28^2^	0.039^2^
	W	1624	0.37^2^	0.39^2^	0.041^2^	−0.12^2^	<0.001^2^	0.31^2^	<0.001^2^	0.37^2^	0.94	0.34^2^	0.46
BMI	M	360	0.00	0.05	0.06	−0.15^2^	0.05	−0.01	0.81	0.11^2^	0.003^2^	0.03	0.27
	W	451	−0.02	0.02	0.035^2^	−0.11^2^	0.17	0.00	0.46	0.03	0.11	0.02	0.42
MUAC	M	402	0.18^2^	0.25^2^	0.05	−0.15^2^	<0.001^2^	0.22^2^	0.38	0.17^2^	0.67	0.13^2^	0.46
	W	517	0.20^2^	0.30^2^	<0.001^2^	−0.29^2^	<0.001^2^	0.22^2^	0.21	0.23^2^	0.26	0.12^2^	0.17
Hemoglobin	M	495	0.14^2^	0.19^2^	0.013^2^	−0.15^2^	<0.001^2^	0.24^2^	<0.001^2^	0.25^2^	<0.001^2^	0.15^2^	0.95
	W	554	0.13^2^	0.17^2^	0.038^2^	−0.12^2^	<0.001^2^	0.19^2^	0.006^2^	0.16^2^	0.63	0.12^2^	0.92

1 AHEI-2010, Alternative Healthy Eating Index–2010; GDQS, Global Diet Quality Score; GDQS−, GDQS negative submetric; GDQS+, GDQS positive submetric; M, men; MDD-W, Minimum Dietary Diversity–Women; MUAC, midupper arm circumference; ONA, energy-adjusted continuous measure of overall nutrient adequacy (number of adequate nutrients out of 8); PDQS, Prime Diet Quality Score; W, women.

2 Indicates statistically significant (*P* < 0.05) correlations (*r*) and Wolfe tests comparing metric-outcome correlations for the GDQS compared with other metrics (*P*-difference). Correlations for iron exclude participants from Ethiopia.

We observed moderate correlations between the GDQS and energy-adjusted overall nutrient adequacy (the number of nutrients, out of 8, consumed in adequate amounts) of 0.34 in men and 0.37 in women (*P* < 0.05) (Table 1). The GDQS+ was more strongly correlated with energy-adjusted overall nutrient adequacy than the GDQS in men (*r* = 0.38; Wolfe test *P* for difference with GDQS <0.001) and women (*r* = 0.39; *P*-difference = 0.041), the PDQS-like metric was less strongly correlated in men (*r* = 0.25; *P*-difference <0.001) and women (*r* = 0.31; *P*-difference <0.001), correlations with the MDD-W did not significantly differ in men (*r* = 0.37; *P*-difference = 0.13) or women (*r* = 0.37; *P*-difference = 0.94), and the AHEI-2010 was less strongly correlated in men (*r* = 0.28; *P*-difference = 0.039) whereas the correlation did not significantly differ in women (*r* = 0.34; *P*-difference = 0.46). The GDQS−, for which higher scores indicate less consumption of unhealthy foods, was negatively correlated with energy-adjusted overall nutrient adequacy in men (*r* = −0.15) and women (*r* = −0.12) (*P* < 0.05). Comparisons of correlations between the GDQS and other diet metrics compared with individual energy-adjusted nutrients are presented in Table 1.

Correlations between the GDQS and energy-adjusted nutrient intakes and overall nutrient adequacy varied by village (**Supplemental Table 6**). In the total population of men and the total population of women, adjustment for village attenuated most correlations (correlation between the GDQS and energy-adjusted overall nutrient adequacy decreased from 0.34 to 0.26 in men, and from 0.37 to 0.27 in women, but remained significant in both sexes, *P* < 0.05) (**Supplemental Table 7**).

In age-adjusted regression models in both men and women, the GDQS was significantly (*P*-trend across quintiles <0.05) associated with higher nutrient adequacy [quintile (Q) 5–Q1 range in estimated marginal means for men: 3.66–4.66 adequate nutrients (out of 8); women: 3.97–5.33], higher MUAC (Q5–Q1 range: 24.25–25.52 cm in men and 24.05–25.83 in women), higher hemoglobin (Q5–Q1 range: 12.30–13.55 g/dL in men and 10.42–11.86 in women), lower odds of low MUAC (Q5 compared with Q1 OR in men: 0.44, 95% CI: 0.22, 0.85; women: 0.57, 95% CI: 0.31, 1.03), and lower odds of anemia (Q5 compared with Q1 OR in men: 0.56, 95% CI: 0.32, 0.98; women: 0.60, 95% CI: 0.35, 1.01) (**Table 2**).

**TABLE 2:** Age-adjusted associations between the GDQS (scored using FFQ data) and continuous and categorical diet quality outcomes in rural sub-Saharan African adults^1^

Outcome	Statistic	Sex	*n*	Cases, *n*	GDQS quintile 1	GDQS quintile 2	GDQS quintile 3	GDQS quintile 4	GDQS quintile 5	Per 1 SD	*P*-trend
ONA (#)	EMM (95% CI)	M	1547		3.66 (3.53, 3.79)	3.86 (3.73, 3.98)	4.27 (4.14, 4.40)	4.56 (4.43, 4.69)	4.66 (4.53, 4.79)	0.38 (0.32, 0.44)	<0.001^2^
		F	1624		3.97 (3.83, 4.11)	4.56 (4.40, 4.71)	5.26 (5.11, 5.41)	5.61 (5.46, 5.76)	5.33 (5.17, 5.48)	0.51 (0.45, 0.58)	<0.001^2^
ONA <4	OR (95% CI)	M	1547	539	REF	0.63 (0.46, 0.86)	0.36 (0.26, 0.50)	0.22 (0.15, 0.31)	0.18 (0.13, 0.26)	0.51 (0.45, 0.57)	<0.001^2^
		F	1624	417	REF	0.41 (0.30, 0.56)	0.16 (0.11, 0.22)	0.08 (0.05, 0.12)	0.09 (0.06, 0.13)	0.35 (0.30, 0.40)	<0.001^2^
BMI, kg/m^2^	EMM (95% CI)	M	360		20.50 (19.92, 21.09)	20.34 (19.63, 21.06)	20.80 (20.11, 21.48)	20.62 (19.84, 21.39)	20.45 (19.67, 21.22)	−0.01 (−0.31, 0.29)	0.89
		F	451		21.34 (20.55, 22.13)	21.95 (21.09, 22.80)	22.31 (21.49, 23.12)	21.96 (21.19, 22.73)	21.61 (20.74, 22.48)	0.08 (−0.28, 0.45)	0.68
BMI <18.5	OR (95% CI)	M	360	88	REF	1.18 (0.59, 2.36)	0.67 (0.31, 1.37)	0.93 (0.43, 1.97)	0.89 (0.41, 1.87)	0.95 (0.75, 1.21)	0.59
		F	451	65	REF	0.90 (0.40, 2.01)	0.55 (0.22, 1.30)	0.82 (0.38, 1.79)	0.84 (0.36, 1.91)	1.00 (0.76, 1.30)	0.64
BMI ≥25	OR (95% CI)	M	360	24	REF	1.55 (0.47, 5.18)	1.13 (0.31, 3.89)	0.85 (0.17, 3.37)	1.20 (0.29, 4.38)	0.99 (0.65, 1.47)	0.87
		F	451	75	REF	1.31 (0.57, 3.02)	1.46 (0.66, 3.26)	1.64 (0.77, 3.60)	1.05 (0.43, 2.50)	1.06 (0.82, 1.36)	0.74
MUAC, cm	EMM (95% CI)	M	402		24.25 (23.52, 24.98)	24.02 (23.17, 24.86)	25.06 (24.22, 25.90)	25.53 (24.56, 26.51)	25.52 (24.52, 26.53)	0.50 (0.11, 0.89)	0.005^2^
		F	517		24.05 (23.36, 24.73)	23.59 (22.89, 24.29)	24.85 (24.12, 25.58)	26.20 (25.43, 26.98)	25.83 (24.97, 26.69)	0.86 (0.52, 1.20)	<0.001^2^
Low MUAC	OR (95% CI)	M	402	192	REF	0.78 (0.43, 1.42)	0.53 (0.29, 0.95)	0.39 (0.20, 0.75)	0.44 (0.22, 0.85)	0.70 (0.56, 0.87)	0.002^2^
		F	517	234	REF	1.50 (0.89, 2.53)	1.22 (0.72, 2.06)	0.41 (0.23, 0.73)	0.57 (0.31, 1.03)	0.75 (0.62, 0.90)	<0.001^2^
Hemoglobin, g/dL	EMM (95% CI)	M	495		12.30 (11.86, 12.75)	12.74 (12.24, 13.25)	13.25 (12.78, 13.71)	13.01 (12.51, 13.51)	13.55 (13.03, 14.07)	0.41 (0.20, 0.62)	<0.001^2^
		F	554		10.42 (10.02, 10.82)	11.01 (10.52, 11.49)	11.27 (10.83, 11.71)	11.68 (11.26, 12.10)	11.86 (11.41, 12.31)	0.52 (0.33, 0.71)	<0.001^2^
Anemia	OR (95% CI)	M	495	220	REF	0.79 (0.45, 1.36)	0.67 (0.39, 1.13)	0.66 (0.38, 1.15)	0.56 (0.32, 0.98)	0.79 (0.66, 0.95)	0.041^2^
		F	554	316	REF	0.99 (0.57, 1.73)	0.99 (0.58, 1.67)	0.72 (0.44, 1.20)	0.60 (0.35, 1.01)	0.84 (0.71, 1.00)	0.026^2^

1 GDQS, Global Diet Quality Score; EMM, estimated marginal mean; MUAC, midupper arm circumference; ONA, energy-adjusted continuous measure of overall nutrient adequacy (number of adequate nutrients out of 8); REF, reference.

2 Indicates statistically significant (*P* < 0.05) linear trends across metric quintiles.

Similar to the GDQS, the GDQS+, PDQS-like metric, MDD-W, and AHEI-2010 were also associated with higher overall nutrient adequacy and hemoglobin in men and women in age-adjusted models; the GDQS+, PDQS-like metric, and MDD-W were also associated with lower odds of anemia in men and women (whereas the AHEI-2010 was only associated in women); and the GDQS+ and PDQS-like metric were further associated with lower odds of low MUAC in men and women (whereas the MDD-W was associated in women and only marginally associated in men, *P* = 0.07). Statistical comparisons of performance between metrics were nonsignificant (*P* ≥ 0.05) except that the GDQS+ outperformed the PDQS-like metric in predicting overall nutrient adequacy (*P* < 0.05) (**Table 3**; **Supplemental Tables 8** and **9**). **Figure 1** shows age-adjusted associations between the GDQS and MDD-W compared with overall nutrient adequacy, low MUAC, and anemia in men and women.

**TABLE 3:** Comparison of age-adjusted associations between the GDQS and other diet metrics compared with selected diet quality outcomes in rural sub-Saharan African adults^1^

						GDQS		Comparison metric		
Comparison metric	Outcome	Statistic	Sex	*n*	Cases, *n*	Per 1 SD	*P*-trend	Per 1 SD	*P*-trend	*P*-difference
GDQS+	Anemia	OR (95% CI)	M	495	220	0.79 (0.66, 0.95)	0.041	0.71 (0.59, 0.85)	0.006^2^	0.17
			F	554	316	0.84 (0.71, 1.00)	0.026	0.82 (0.68, 0.97)	0.044^2^	0.66
	BMI <18.5 kg/m^2^	OR (95% CI)	M	360	88	0.95 (0.75, 1.21)	0.585	0.86 (0.66, 1.10)	0.25	0.21
			F	451	65	1.00 (0.76, 1.30)	0.644	0.84 (0.63, 1.10)	0.37	0.24
	BMI ≥25	OR (95% CI)	M	360	24	0.99 (0.65, 1.47)	0.874	1.21 (0.78, 1.86)	0.56	0.29
			F	451	75	1.06 (0.82, 1.36)	0.741	1.14 (0.88, 1.48)	0.29	0.25
	Low MUAC	OR (95% CI)	M	402	192	0.70 (0.56, 0.87)	0.002	0.63 (0.50, 0.79)	0.005^2^	0.90
			F	517	234	0.75 (0.62, 0.90)	0.000	0.60 (0.49, 0.72)	<0.001^2^	0.05
	ONA (#)	EMM (95% CI)	M	1547		0.38 (0.32, 0.44)	0.000	0.43 (0.37, 0.49)	<0.001^2^	0.08
			F	1624		0.51 (0.45, 0.58)	0.000	0.55 (0.48, 0.62)	<0.001^2^	0.15
GDQS−	Anemia	OR (95% CI)	M	495	220	0.79 (0.66, 0.95)	0.041	1.35 (1.12, 1.63)	0.031^2^	0.06
			F	554	316	0.84 (0.71, 1.00)	0.026	1.08 (0.91, 1.30)	0.06	0.08
	BMI <18.5	OR (95% CI)	M	360	88	0.95 (0.75, 1.21)	0.585	1.30 (1.00, 1.70)	0.19	0.31
			F	451	65	1.00 (0.76, 1.30)	0.644	1.56 (1.17, 2.10)	0.002^2^	0.16
	BMI ≥25	OR (95% CI)	M	360	24	0.99 (0.65, 1.47)	0.874	0.62 (0.41, 0.95)	0.08	0.20
			F	451	75	1.06 (0.82, 1.36)	0.741	0.83 (0.63, 1.07)	0.012^2^	0.33
	Low MUAC	OR (95% CI)	M	402	192	0.70 (0.56, 0.87)	0.002	1.26 (1.02, 1.56)	0.09	0.07
			F	517	234	0.75 (0.62, 0.90)	0.000	1.78 (1.47, 2.17)	<0.001^2^	0.038^2^
	ONA (#)	EMM (95% CI)	M	1547		0.38 (0.32, 0.44)	0.000	−0.18 (−0.24, −0.11)	<0.001^2^	0.001^2^
			F	1624		0.51 (0.45, 0.58)	0.000	−0.20 (−0.27, −0.12)	<0.001^2^	<0.001^2^
PDQS-like metric	Anemia	OR (95% CI)	M	495	220	0.79 (0.66, 0.95)	0.041	0.65 (0.54, 0.78)	0.002^2^	0.13
			F	554	316	0.84 (0.71, 1.00)	0.026	0.78 (0.66, 0.92)	0.032^2^	0.69
	BMI <18.5	OR (95% CI)	M	360	88	0.95 (0.75, 1.21)	0.585	1.04 (0.81, 1.33)	0.60	0.24
			F	451	65	1.00 (0.76, 1.30)	0.644	0.94 (0.73, 1.23)	0.54	0.23
	BMI ≥25	OR (95% CI)	M	360	24	0.99 (0.65, 1.47)	0.874	1.10 (0.71, 1.69)	0.53	0.22
			F	451	75	1.06 (0.82, 1.36)	0.741	1.04 (0.81, 1.34)	0.82	0.76
	Low MUAC	OR (95% CI)	M	402	192	0.70 (0.56, 0.87)	0.002	0.66 (0.53, 0.83)	0.028^2^	0.55
			F	517	234	0.75 (0.62, 0.90)	0.000	0.71 (0.59, 0.85)	0.003^2^	0.44
	ONA (#)	EMM (95% CI)	M	1547		0.38 (0.32, 0.44)	0.000	0.30 (0.24, 0.36)	0.000^2^	0.06
			F	1624		0.51 (0.45, 0.58)	0.000	0.46 (0.39, 0.53)	0.000^2^	0.08
MDD-W	Anemia	OR (95% CI)	M	495	220	0.79 (0.66, 0.95)	0.041	0.63 (0.53, 0.76)	0.001^2^	0.10
			F	554	316	0.84 (0.71, 1.00)	0.026	0.75 (0.62, 0.89)	0.031^2^	0.397
	BMI <18.5	OR (95% CI)	M	360	88	0.95 (0.75, 1.21)	0.585	0.86 (0.68, 1.09)	0.37	0.36
			F	451	65	1.00 (0.76, 1.30)	0.644	0.91 (0.71, 1.17)	0.41	0.47
	BMI ≥25	OR (95% CI)	M	360	24	0.99 (0.65, 1.47)	0.874	1.44 (0.94, 2.26)	0.22	0.23
			F	451	75	1.06 (0.82, 1.36)	0.741	1.24 (0.97, 1.60)	0.13	0.15
	Low MUAC	OR (95% CI)	M	402	192	0.70 (0.56, 0.87)	0.002	0.65 (0.52, 0.81)	0.07	0.51
			F	517	234	0.75 (0.62, 0.90)	0.000	0.71 (0.59, 0.85)	<0.001^2^	0.20
	ONA (#)	EMM (95% CI)	M	1547		0.38 (0.32, 0.44)	0.000	0.45 (0.39, 0.50)	<0.001^2^	0.39
			F	1624		0.51 (0.45, 0.58)	0.000	0.58 (0.51, 0.64)	<0.001^2^	0.20
AHEI-2010	Anemia	OR (95% CI)	M	495	220	0.79 (0.66, 0.95)	0.041	0.74 (0.61, 0.88)	0.002^2^	0.35
			F	554	316	0.84 (0.71, 1.00)	0.026	0.86 (0.73, 1.00)	0.14	0.70
	BMI <18.5	OR (95% CI)	M	360	88	0.95 (0.75, 1.21)	0.585	1.05 (0.83, 1.32)	0.48	0.42
			F	451	65	1.00 (0.76, 1.30)	0.644	1.02 (0.79, 1.32)	0.819	0.44
	BMI ≥25	OR (95% CI)	M	360	24	0.99 (0.65, 1.47)	0.874	1.47 (0.98, 2.23)	0.12	0.34
			F	451	75	1.06 (0.82, 1.36)	0.741	1.19 (0.94, 1.53)	0.19	0.36
	Low MUAC	OR (95% CI)	M	402	192	0.70 (0.56, 0.87)	0.002	0.72 (0.58, 0.89)	0.003^2^	0.68
			F	517	234	0.75 (0.62, 0.90)	0.000	0.80 (0.67, 0.96)	0.06	0.39
	ONA (#)	EMM (95% CI)	M	1547		0.38 (0.32, 0.44)	0.000	0.32 (0.26, 0.38)	<0.001^2^	0.20
			F	1624		0.51 (0.45, 0.58)	0.000	0.50 (0.43, 0.57)	<0.001^2^	0.31

1 Excluded from this table are outcomes categorized as lower clinical relevance (the binary measure of energy-adjusted overall nutrient inadequacy and continuous BMI, MUAC, and hemoglobin); refer to Supplemental Table 9 for expanded results. AHEI-2010, Alternative Healthy Eating Index–2010; EMM, estimated marginal mean; GDQS, Global Diet Quality Score; GDQS−, GDQS negative submetric; GDQS+, GDQS positive submetric; MDD-W, Minimum Dietary Diversity–Women; MUAC, midupper arm circumference; ONA, energy-adjusted continuous measure of overall nutrient adequacy (number of adequate nutrients out of 8); PDQS, Prime Diet Quality Score.

2 Indicates statistically significant (*P* < 0.05) linear trends across metric quintiles (*P*-trend) and Wald tests comparing trends between the GDQS and other metrics (*P*-difference).

The GDQS− was the only metric associated with a lower odds of overweight BMI in age-adjusted models, in women only (Q5 compared with Q1 OR: 0.53, 95% CI: 0.21, 1.19) (*P* < 0.05) (Table 3). The GDQS− was also the only metric associated with a higher odds of underweight BMI, in women only (Q5 compared with Q1 OR: 2.99, 95% CI: 1.33, 6.84) (*P* < 0.05).

Expanded correlation statistics and comparisons are presented in **Supplemental Table 10**, and expanded regression statistics and model comparisons are presented in Supplemental Tables 8 and 9, respectively. A summary of significant results of regression analyses is presented in **Supplemental Table 11**.

## Discussion

In this secondary analysis of rural men and nonpregnant nonlactating women of reproductive age in 10 African countries, we found modest positive correlations between the GDQS and energy-adjusted intakes of fiber, folate, monounsaturated fat, polyunsaturated fat, protein, and vitamin A. GDQS-nutrient correlations were generally stronger in women than men, and varied by village. Controlling for village attenuated most GDQS-nutrient correlations, indicating that between-village variation in diet quality is an important determinant of nutrient intakes and adequacy in this population.

In age-adjusted regression models, the GDQS was positively associated with overall nutrient adequacy in men and women [consistent with findings of parallel GDQS evaluations in men and women in China and Ethiopia, and women in India and Mexico (61–64)], reduced odds of low MUAC in men and women [consistent with findings in Ethiopian and Indian women (61, 64)], and reduced odds of anemia in men and women [consistent with findings in Ethiopian women (61)]. Regression models did not find major differences in the performance of the GDQS, GDQS+, a PDQS-like metric, and MDD-W in analyses of anemia or low MUAC (all 4 metrics were predictive of lower odds of these outcomes in men and women, though the MDD-W was marginally associated with low MUAC in men), whereas the AHEI-2010 was not associated with either outcome in men or women. The GDQS− was inversely associated with overweight/obesity in women (other metrics were not associated).

In prior secondary analysis of the MDD-W using quantitative 24HR data from nonpregnant nonlactating women of reproductive age in diverse resource-poor settings (including rural populations in 3 African countries), Pearson correlations between the MDD-W (scored as a continuous variable from 0 to 10) and energy-adjusted mean probability of adequacy of 11 nutrients ranged from 0.29 (rural Uganda), to 0.31 (rural Mozambique), to 0.48 (rural Burkina Faso) (49). Our current analysis found a correlation of 0.37 for both the MDD-W and GDQS compared with overall nutrient adequacy in the total population of women across 10 countries, although comparisons between these studies are complicated by differences in the countries, dietary instruments, nutrient adequacy variable, and analytical approaches involved, including how the aggregate variable of nutrient adequacy was defined. Only 1 prior study in Africa, involving secondary analysis of quantitative 24HR data from 7533 pregnant Tanzanian women, has evaluated the comparative performance of the MDD-W and the earlier PDQS (upon which the GDQS is based) in predicting pregnancy outcomes, and found the PDQS to be inversely associated with preterm birth, low birth weight, and fetal loss, whereas the MDD-W was inversely associated with small for gestational age (48).

In the current analysis, the GDQS was more strongly correlated than the MDD-W with energy-adjusted protein intake in women (likely reflecting the expanded list of animal-source food groups in the GDQS) and less negatively correlated than other metrics with zinc in both men and women. The negative correlation between the MDD-W and zinc was driven mainly by plant-source components, namely, vitamin A–rich fruits and vegetables, other vegetables, and other fruits; fruit and vegetable components also drove the negative correlation between the AHEI-2010 and zinc. Negative correlations with zinc (as well as calcium and iron) reflect the fact that, on an energy-adjusted basis, total consumption of fruits and vegetables (which are scored positively in all diet metrics) correlated negatively with total consumption of mineral-rich animal-source foods in this population (partial correlation controlling for energy was −0.14 in men and −0.17 in women; *P* < 0.001). Whereas GDQS fruit and vegetable groups were also somewhat negatively correlated with mineral intakes, these groups are more disaggregated and numerous in the GDQS, which helps to moderate the extent to which any particular group might drive metric-outcome associations (when computing the GDQS, this aspect of the metric may also mitigate the influence of measurement error in food consumption).

Conversely, the AHEI-2010 was more strongly correlated among metrics with energy-adjusted intake of polyunsaturated fat (reflecting the inclusion of polyunsaturated fat as an AHEI-2010 scoring component), whereas the MDD-W exhibited stronger correlations than the GDQS with monounsaturated fat in women (driven mainly by the MDD-W nuts and seeds component), vitamin A in men and women (driven by the vitamin A–rich fruits and vegetables component), and fiber in women (driven by pulses, dark green leafy vegetables, and other vegetables and fruits). In settings where dietary diversity is very low, the MDD-W (which employs fewer food groups, no negatively scored groups, and a simpler scoring approach) may be similarly sensitive to nutrient adequacy (in such settings, the relative complexity of the GDQS may not necessarily add predictive value for assessing nutrient adequacy). In a separate evaluation of a particularly resource-poor context (a predominantly rural population of Ethiopian men and women), we found the MDD-W to be more sensitive than the GDQS in capturing overall nutrient adequacy (61); however, this was not observed in evaluating the GDQS in the current analysis, or in China, India, or Mexico (62–64).

In the current analysis, we observed higher correlations for the GDQS+ than the GDQS with energy-adjusted fiber, folate, and vitamin A intakes. This is due to the inclusion in the GDQS of negatively scored food groups that also contribute some dietary nutrients (white roots and tubers, and refined grains in particular are significant sources of some nutrients in rural SSA given their volume of consumption, and scoring these foods negatively in the GDQS could somewhat attenuate GDQS-nutrient correlations). The GDQS partly addresses this by giving positive scores to red meat and high fat dairy up until specific consumption thresholds, after which these groups receive zero points. The inclusion of negatively scored food groups in the GDQS is intended to help the metric capture diet-related NCD risk more sensitively than the GDQS+ and serve as a measure of overall diet quality (whereas the GDQS+ explicitly captures the contribution of healthy foods to diet quality). Importantly, we did not observe differences in associations between the GDQS and GDQS+ compared with overall nutrient adequacy, low MUAC, or anemia, indicating that inclusion of negatively scored foods did not impair the ability of the GDQS to capture these key indicators of nutrient adequacy.

We also observed strongly negative correlations between the GDQS− and energy-adjusted fiber, folate, iron, saturated fat, and vitamin A intakes, owing to this submetric's sole inclusion of negatively scored foods (with the exception of moderate red meat and high fat dairy consumption) and the fact that these foods do contribute some dietary nutrients. The GDQS− explicitly captures the extent to which unhealthy foods contribute to dietary nutrient intake, but does not intend to capture undernutrition outcomes (and was unfavorably associated with overall nutrient adequacy, low MUAC, underweight BMI, and anemia in the current study). However, that the GDQS− was the only metric inversely associated with overweight highlights its role in capturing diet-related NCD risk, and further supports the inclusion of negatively scored components in the GDQS to allow more holistic measurement of diet quality (and which we have found add value to the metric in capturing NCD outcomes in parallel evaluations of the GDQS in China, Mexico, and the United States) (63, 65–67).

This study has many strengths. These include broad inclusion of villages across 10 SSA countries, use of FFQs specifically developed for each country, and use of country-specific food composition data. This study also has limitations. First, although we derived standard portion sizes for as many SSA foods as possible through analysis of open-ended 24HR data from rural populations, error might arise due to differences in diet between the sample demographics and countries represented by the 24HR surveys (which included rural women of reproductive age in 3 countries, and men and nonpregnant nonlactating women of reproductive age in 1 country) and the Millennium Villages (which included rural men and nonpregnant nonlactating women in 10 countries). A second limitation is that the cross-sectional nature of this study prevented us from inferring causal relations between diet quality metrics and outcomes. A third important limitation of this study is that it does not provide evidence to support that GDQS is a suitable metric for capturing NCD risk. This is due to the limited number of NCD-related outcomes in the current study, of which only fatty acid and fiber intakes, and overweight/obesity were available (furthermore, the low prevalence of overweight, 7% in men and 17% in women, diminished statistical power to derive associations for that outcome).

In conclusion, the GDQS is evidently a useful measure of nutrient adequacy, low MUAC, and anemia in SSA men and women. Based on comparison with the MDD-W, the differentiation of healthy- and unhealthy-scoring components by the GDQS did not appear to compromise its ability to sensitively capture nutrient adequacy–related outcomes. This is important given the emerging double burden of undernutrition and NCDs in SSA, and the need for an easily operationalized metric that captures both nutrient adequacy and diet-related NCD risk. Although we have found the MDD-W to be sensitive to nutrient adequacy–related outcomes in this and other settings (61–64), and somewhat simpler to collect, its lack of differentiation between healthy and unhealthy food groups has limited its ability to capture NCD risks in other settings (63, 65–67). Nonetheless, given the lack of data on NCD outcomes in this study, prospective studies are warranted to compare performance of both these metrics and the AHEI in relation to NCD outcomes in SSA.

## Supplementary Material

nxab161_Supplemental_File

## Notes

Funding for the research was provided by FHI Solutions, recipient of a Bill & Melinda Gates Foundation grant to support Intake – Center for Dietary Assessment.

Author disclosures: TTF is an Associate Editor for the Journal of Nutrition and played no role in the Journal’s evaluation of the manuscript. All other authors report no conflicts of interest.

Publication costs for this supplement were funded by the Bill & Melinda Gates Foundation in conjunction with FHI Solutions, recipient of a Bill & Melinda Gates Foundation grant to support Intake – Center for Dietary Assessment. The opinions expressed in this publication are those of the authors and are not attributable to the sponsors or the publisher, Editor, or Editorial Board of *The Journal of Nutrition*. The Supplement Coordinators for the supplement publication were Megan Deitchler, Intake – Center for Dietary Assessment at FHI Solutions, Washington, DC; and Sabri Bromage, Harvard T.H. Chan School of Public Health, Boston, MA.

Supplemental Tables 1–11 are available from the “Supplementary data” link in the online posting of the article and from the same link in the online table of contents available at https://academic.oup.com/jn.

The GDQS research initiative was launched by Intake – Center for Dietary Assessment. The research was led by Harvard T.H. Chan School of Public Health, Department of Nutrition and carried out in collaboration with researchers at the National Public Health Institute (INSP), Mexico. Funding for the research was provided by FHI Solutions, recipient of a Bill & Melinda Gates Foundation grant to support Intake – Center for Dietary Assessment.

Abbreviations used: AHEI-2010, Alternative Healthy Eating Index–2010; EAR, estimated average requirement; FCT, food composition table; GDQS, Global Diet Quality Score; GDQS−, GDQS negative submetric; GDQS+, GDQS positive submetric; MDD-W, Minimum Dietary Diversity–Women; MUAC, midupper arm circumference; MVP, Millennium Villages Project; NCD, noncommunicable disease; PDQS, Prime Diet Quality Score; Q, quintile; SSA, sub-Saharan Africa; 24HR, 24-h recall.

## References

[1] Muyonga JH , Nansereko S, Steenkamp I, Manley M, Okoth JK. Traditional African foods and their potential to contribute to health and nutrition. In: Shekhar HU, Howlader ZH, Kabir Y, editors. Exploring the nutrition and health benefits of functional foods. IGI Global; 2017. p. 320–46.

[2] Miracle MP . The introduction and spread of maize in Africa. J Afr Hist. 1965;6:39–55.

[3] Hollinger F , Staatz JM. Agricultural growth in West Africa. Market and policy drivers. Rome, Italy: FAO, African Development Bank, ECOWAS; 2015.

[4] Food and Agriculture Organization. Percentage of Undernourished people by region in 2000 and 2019. [Internet]. [cited 2021 February 27]. Available from: http://www.fao.org/sustainable-development-goals/indicators/211/en/.

[5] Holmes MD , Dalal S, Sewram V, Diamond MB, Adebamowo SN, Ajayi IO, Adebamowo C, Chiwanga FS, Njelekela M, Laurence C et al. Consumption of processed food dietary patterns in four African populations. Public Health Nutr. 2018;21(8):1529–37. 29388531 10.1017/S136898001700386X PMC10261518

[6] Reardon T , Tschirley D, Liverpool-Tasie LSO, Awokuse T, Fanzo J, Minten B, Vos R, Dolislager M, Sauer C, Dhar R et al. The processed food revolution in African food systems and the double burden of malnutrition. Glob Food Sec. 2021;28:100466. 10.1016/j.gfs.2020.100466 PMC8049356 33868911

[7] Food and Agriculture Organization. FAOSTAT3 system. [Internet]. [cited 2021 February 27]. Available from:http://www.fao.org/faostat/.

[8] Muthayya S , Rah JH, Sugimoto JD, Roos FF, Kraemer K, Black RE. The global hidden hunger indices and maps: an advocacy tool for action. PLoS One. 2013;8(6):e67860. 23776712 10.1371/journal.pone.0067860 PMC3680387

[9] UNICEF, WHO, The World Bank. Joint child malnutrition estimates: levels and trends. [Internet]. [cited 2021 February 27]. Available from: https://www.who.int/nutgrowthdb/estimates/en/.

[10] Bosu WK . An overview of the nutrition transition in West Africa: implications for non-communicable diseases. Proc Nutr Soc. 2015;74(4):466–77. 25529539 10.1017/S0029665114001669

[11] Steyn NP , McHiza ZJ. Obesity and the nutrition transition in sub-Saharan Africa. Ann N Y Acad Sci. 2014;1311:88–101. 24725148 10.1111/nyas.12433

[12] Nnyepi MS , Gwisai N, Lekgoa M, Seru T. Evidence of nutrition transition in Southern Africa. Proc Nutr Soc. 2015;74(4):478–86. 25686639 10.1017/S0029665115000051

[13] Development Initiatives Poverty Research. 2020 Global Nutrition Report: action on equity to end malnutrition. Bristol, United Kingdom: Development Initiatives; 2020.

[14] Popkin BM , Corvalan C, Grummer-Strawn LM. Dynamics of the double burden of malnutrition and the changing nutrition reality. Lancet. 2020;395(10217):65–74. 31852602 10.1016/S0140-6736(19)32497-3 PMC7179702

[15] NCD Risk Factor Collaboration (NCD-RisC). Rising rural body-mass index is the main driver of the global obesity epidemic in adults. Nature. 2019;569(7755):260–4. 31068725 10.1038/s41586-019-1171-x PMC6784868

[16] International Health Metrics and Evaluation. GBD compare [Internet]. [cited 2021 February 27]. Available from: https://vizhub.healthdata.org/gbd-compare/.

[17] de Ramirez SS , Enquobahrie DA, Nyadzi G, Mjungu D, Magombo F, Ramirez M, Sachs SE, Willett W. Prevalence and correlates of hypertension: a cross-sectional study among rural populations in sub-Saharan Africa. J Hum Hypertens. 2010;24(12):786–95. 20220771 10.1038/jhh.2010.14

[18] Noubiap JJ , Bigna JJ, Nansseu JR, Nyaga UF, Balti EV, Echouffo-Tcheugui JB, Kengne AP. Prevalence of dyslipidaemia among adults in Africa: a systematic review and meta-analysis. Lancet Glob Health. 2018;6(9):e998–e1007. 30103999 10.1016/S2214-109X(18)30275-4

[19] Bosu WK , Reilly ST, Aheto JMK, Zucchelli E. Hypertension in older adults in Africa: a systematic review and meta-analysis. PLoS One. 2019;14(4):e0214934. 30951534 10.1371/journal.pone.0214934 PMC6450645

[20] Atun R , Davies JI, Gale EAM, Bärnighausen T, Beran D, Kengne AP, Levitt NS, Mangugu FW, Nyirenda MJ, Ogle GD et al. Diabetes in sub-Saharan Africa: from clinical care to health policy. Lancet Diabetes Endocrinol. 2017;5(8):622–67. 28688818 10.1016/S2213-8587(17)30181-X

[21] GBD 2017 Diet Collaborators. Health effects of dietary risks in 195 countries, 1990–2017: a systematic analysis for the Global Burden of Disease Study 2017. Lancet. 2019;393(10184):1958–72. 30954305 10.1016/S0140-6736(19)30041-8 PMC6899507

[22] Imamura F , Micha R, Khatibzadeh S, Fahimi S, Shi P, Powles J, Mozaffarian D; Global Burden of Diseases Nutrition and Chronic Diseases Expert Group (NutriCoDE). Dietary quality among men and women in 187 countries in 1990 and 2010: a systematic assessment. Lancet Glob Health. 2015;3(3):e132–42. 25701991 10.1016/S2214-109X(14)70381-X PMC4342410

[23] Chaudhary A , Gustafson D, Mathys A. Multi-indicator sustainability assessment of global food systems. Nat Commun. 2018;9(1):848. 29487286 10.1038/s41467-018-03308-7 PMC5829192

[24] Melaku YA , Gill TK, Taylor AW, Appleton SL, Gonzalez-Chica D, Adams R, Achoki T, Shi Z, Renzaho A. Trends of mortality attributable to child and maternal undernutrition, overweight/obesity and dietary risk factors of non-communicable diseases in sub-Saharan Africa, 1990–2015: findings from the Global Burden of Disease Study 2015. Public Health Nutr. 2019;22(5):827–40. 30509334 10.1017/S1368980018002975 PMC10260606

[25] Wang DD , Li Y, Afshin A, Springmann M, Mozaffarian D, Stampfer MJ, Hu FB, Murray CJL, Willett WC. Global improvement in dietary quality could lead to substantial reduction in premature death. J Nutr. 2019;149(6):1065–74. 31049577 10.1093/jn/nxz010 PMC6543201

[26] Mbogori T , Kimmel K, Zhang M, Kandiah J, Wang Y. Nutrition transition and double burden of malnutrition in Africa: a case study of four selected countries with different social economic development. AIMS Public Health. 2020;7(3):425–39. 32968668 10.3934/publichealth.2020035 PMC7505783

[27] Global Dietary Database. Survey availability information. [Internet]. [cited 2021 February 27]. Available from:https://www.globaldietarydatabase.org/our-data/data-visualizations/survey-availability-information.

[28] Agriculture and Food Systems Institute. World nutrient databases for dietary studies (WNDDS). [Internet]. [cited 2021 February 27]. Available from: https://foodsystems.org/resources/wndds/.

[29] Bromage S , Batis C, Bhupathiraju SN, Fawzi WW, Fung TT, Li Y, Deitchler M, Angulo E, Birk N, Castellanos-Gutiérrez A et al. Development and validation of a novel food-based global diet quality score (GDQS). J Nutr. 2021;151(Suppl 10):75S–92S. 10.1093/jn/nxab244 PMC8542096 34689200

[30] Sanchez P , Palm C, Sachs J, Denning G, Flor R, Harawa R, Jama B, Kiflemariam T, Konecky B, Kozar R et al. The African Millennium Villages. Proc Natl Acad SciU S A 2007;104(43):16775–80. 17942701 10.1073/pnas.0700423104 PMC2040451

[31] Mitchell S , Gelman A, Ross R, Chen J, Bari S, Huynh UK, Harris MW, Sachs SE, Stuart EA, Feller A et al. The Millennium Villages Project: a retrospective, observational, endline evaluation. Lancet Glob Health. 2018;6(5):e500–13. 29653625 10.1016/S2214-109X(18)30065-2

[32] Leclercq C , Allemand P, Balcerzak A, Branca F, Sousa RF, Lartey A, Lipp M, Quadros VP, Verger P. FAO/WHO GIFT (Global Individual Food consumption data tool): a global repository for harmonised individual quantitative food consumption studies. Proc Nutr Soc. 2019;78(4):484–95. 30816080 10.1017/S0029665119000491

[33] Tadesse AW , Hemler EC, Andersen C, Passarelli S, Worku A, Sudfeld CR, Berhane Y, Fawzi WW. Anemia prevalence and etiology among women, men, and children in Ethiopia: a study protocol for a national population-based survey. BMC Public Health. 2019;19(1):1369. 31651278 10.1186/s12889-019-7647-7 PMC6814127

[34] Hotz C , Abdelrahman L, Sison C, Moursi M, Loechl C. A food composition table for Central and Eastern Uganda. Washington (DC): International Food Policy Research Institute and International Center for Tropical Agriculture; 2012.

[35] Sanusi RA , Olurin A. Portion and serving sizes of commonly consumed foods, in Ibadan, Southwestern Nigeria. Afr J Biomed Res. 2012;15:149–58.

[36] Lukmanji Z , Hertzmark E, Mlingi N, Assey V, Ndossi G, Fawzi WW. Tanzania food composition tables. Dar Es Salaam, Tanzania: Muhimbili University of Health and Allied Sciences and Harvard School of Public Health; 2008.

[37] Food and Agriculture Organization. International network of food data systems (INFOODS). Tables and databases, Africa. [Internet]. [cited 2021 February 27]. Available from:http://www.fao.org/infoods/infoods/tables-and-databases/africa/en/.

[38] NutriSurvey. Country specific databases. [Internet]. [cited 2021 February 27]. Available from: http://www.nutrisurvey.de/.

[39] Hartmann BM , Bell S, Vásquez-Caicedo AL. Bundeslebensmittelschlüssel II 3.1. Karlsruhe, Germany: Federal Research Centre for Nutrition and Food; 2005.

[40] Haytowitz DB . USDA national nutrient database for standard reference, release 24. Washington (DC): United States Department of Agriculture; 2011.

[41] Food and Agriculture Organization. FAO/INFOODS e-learning course on food composition data. [Internet]. [cited 2021 February 27]. Available from:https://elearning.fao.org/course/view.php?id=191.

[42] Bognár A . Tables on weight yield of food and retention factors of food constituents for the calculation of nutrient composition of cooked foods (dishes). Karlsruhe, Germany: Federal Research Centre for Nutrition and Food; 2002.

[43] Matthews RH , Garrison YJ. Agriculture handbook no. 102: food yields summarized by different stages of preparation. Washington (DC): USDA Agricultural Research Service; 1975.

[44] Nutrient Data Laboratory. USDA table of nutrient retention factors. Beltsville (MD): United States Department of Agriculture Agricultural Research Service; 2007.

[45] Showell BA , Williams JR, Duvall M, Howe JC, Patterson KY, Roseland JM, Holden JM. USDA table of cooking yields for meat and poultry. Baltimore (MD): United States Department of Agriculture; 2012.

[46] Fung TT , Isanaka S, Hu FB, Willett WC. International food group-based diet quality and risk of coronary heart disease in men and women. Am J Clin Nutr. 2018;107(1):120–9. 29381797 10.1093/ajcn/nqx015 PMC5972643

[47] Gicevic S , Gaskins AJ, Fung TT, Rosner B, Tobias DK, Isanaka S, Willett WC. Evaluating pre-pregnancy dietary diversity vs. dietary quality scores as predictors of gestational diabetes and hypertensive disorders of pregnancy. PLoS One. 2018;13(4):e0195103. 29614105 10.1371/journal.pone.0195103 PMC5882133

[48] Madzorera I , Isanaka S, Wang M, Msamanga GI, Urassa W, Hertzmark E, Duggan C, Fawzi WW. Maternal dietary diversity and dietary quality scores in relation to adverse birth outcomes in Tanzanian women. Am J Clin Nutr. 2020;112(3):695–706. 32651998 10.1093/ajcn/nqaa172 PMC7458779

[49] WDDP Study Group. Development of a dichotomous indicator for population-level assessment of dietary diversity in women of reproductive age. Curr Dev Nutr. 2017;1(12):cdn.117.001701. 29955691 10.3945/cdn.117.001701 PMC5998796

[50] Chiuve SE , Fung TT, Rimm EB, Hu FB, McCullough ML, Wang M, Stampfer MJ, Willett WC. Alternative dietary indices both strongly predict risk of chronic disease. J Nutr. 2012;142:1009–18. 22513989 10.3945/jn.111.157222 PMC3738221

[51] Willett W , Stampfer MJ. Total energy intake: implications for epidemiologic analyses. Am J Epidemiol. 1986;124(1):17–27. 3521261 10.1093/oxfordjournals.aje.a114366

[52] Institute of Medicine. Dietary reference intakes. The essential guide to nutrient requirements. Washington (DC): National Academies Press; 2006.

[53] Institute of Medicine. Dietary reference intakes: applications in dietary assessment. Washington (DC): National Academies Press; 2000. 25057725

[54] Martin-Prevel Y , Allemand P, Wiesmann D, Arimond M, Ballard T, Deitchler M, Dop M, Kennedy G, Lee WTK, Moursi M. Moving forward on choosing a standard operational indicator of women's dietary diversity. Rome, Italy: Food and Agricultural Organization of the United Nations; 2015.

[55] Allen L , de Benoist B, Dary O, Hurrell Reditors. Guidelines on food fortification with micronutrients. Geneva, Switzerland: World Health Organization; 2004.

[56] International Zinc Nutrition Consultative Group (IZiNCG), Brown KH, Rivera JA, Bhutta Z, Gibson RS, King JC, Lönnerdal B, Ruel MT, Sandtröm B, Wasantwisut E, et al. International Zinc Nutrition Consultative Group (IZiNCG) technical document #1. Assessment of the risk of zinc deficiency in populations and options for its control. Food Nutr Bull 2004;25(1 Suppl 2):S99–203. 18046856

[57] World Health Organization. Haemoglobin concentrations for the diagnosis of anaemia and assessment of severity. Vitamin and Mineral Nutrition Information System. Geneva, Switzerland: WHO; 2011.

[58] WHO Expert Committee on Physical Status. Physical status: the use of and interpretation of anthropometry, report of a WHO Expert Committee. Geneva, Switzerland: WHO; 1995. 8594834

[59] Tang AM , Chung M, Dong K, Wanke C, Bahwere P, Bose K, Chakraborty R, Charlton K, Hong S, Nguyen P et al. Determining a global mid-upper arm circumference cutoff to assess underweight in adults (men and nonpregnant women). Washington (DC): FHI 360/FANTA; 2016.

[60] Wolfe DA . A distribution-free test for related correlation coefficients. Technometrics. 1977;19:507–9.

[61] Bromage S , Andersen CT, Tadesse AW, Passarelli S, Hemler E, Fekadu H, Sudfeld C, Worku A, Berhane H, Batis C et al. The Global Diet Quality Score is associated with higher nutrient adequacy, midupper arm circumference, venous hemoglobin, and serum folate among urban and rural Ethiopian adults. J Nutr. 2021;151(Suppl 10):130S–42S. 10.1093/jn/nxab264 PMC8564694 34689198

[62] Castellanos-Gutiérrez A , Rodríguez-Ramírez S, Bromage S, Fung TT, Li Y, Bhupathiraju SN, Deitchler M, Willett WC, Batis C. Performance of the Global Dietary Quality Score with nutrition and health outcomes in Mexico with 24-hr and FFQ data. J Nutr. 2021;151(Suppl 10):143S–51S. 10.1093/jn/nxab202 PMC8542100 34689195

[63] He Y , Fang Y, Bromage S, Fung TT, Bhupathiraju SN, Batis CB, Deitchler M, Fawzi WW, Stampfer MJ, Hu FB et al. Application of the Global Diet Quality Score in Chinese adults to evaluate the double burden of nutrient inadequacy and metabolic syndrome. J Nutr. 2021;151(Suppl 10):93S–100S. 10.1093/jn/nxab162 PMC8542094 34689199

[64] Matsuzaki M , Birk N, Bromage S, Bowen L, Batis C, Fung TT, Li Y, Stampfer MJ, Deitchler M, Willett WC et al. Validation of Global Diet Quality Score among nonpregnant women of reproductive age in India: Findings from the Andhra Pradesh Children and Parents Study (APCAPS) and the Indian Migration Study (IMS). J Nutr. 2021;151(Suppl 10):101S–9S. 10.1093/jn/nxab217 PMC8564710 34689191

[65] Angulo A , Stern D, Castellanos-Gutiérrez A, Mongue A, Lajous M, Bromage S, Fung TT, Li Y, Bhupathiraju S, Deitchler M et al. Changes in the Global Dietary Quality Score, weight and waist circumference in Mexican women. J Nutr. 2021;151(Suppl 10):152S–61S. 10.1093/jn/nxab171 PMC8542099 34689194

[66] Fung TT , Bromage S, Li Y, Bhupathiraju SN, Batis C, Fawzi WF, Holmes MD, Stampfer MJ, Hu FB, Deitchler M et al. Higher Global Diet Quality Score is associated with less 4-year weight gain in U.S. women. J Nutr. 2021;151(Suppl 10):162S–7S. 10.1093/jn/nxab170 PMC8542092 34689192

[67] Fung TT , Li Y, Bhupathiraju SN, Bromage S, Batis C, Holmes MD, Stampfer MJ, Hu FB, Deitchler M, Willett WC et al. Higher global diet quality score is inversely associated with risk of type 2 diabetes in U.S. women. J Nutr. 2021;151(Suppl 10):168S–75S. 10.1093/jn/nxab195 PMC8542093 34689196

